# Application of the Montgomery Equation in Morphometric Analysis of Tepals: A Case Study of *Liriodendron* × *sinoamericanum*

**DOI:** 10.3390/plants14121861

**Published:** 2025-06-17

**Authors:** Zhuyue Shi, Jinfeng Wang, Guohong Sun, Wenjing Yao, Peijian Shi, Honghua Ruan

**Affiliations:** 1 Southern Modern Forestry Collaborative Innovation Center, College of Ecology and Environment, Nanjing Forestry University, Nanjing 210037, China; shizhuyue@njfu.edu.cn; 2 Bamboo Research Institute, Nanjing Forestry University, Nanjing 210037, China; jfwang@njfu.edu.cn (J.W.); yaowenjing@njfu.edu.cn (W.Y.); 3 College of Forestry, Northwest A&F University, Xianyang 712100, China; 4 College of Forestry and Grassland, Nanjing Forestry University, Nanjing 210037, China; sunguohong@njfu.edu.cn

**Keywords:** Montgomery equation, perianth differentiation and evolution, tepal area, tepal shape

## Abstract

Distinctions between plant perianths are often defined by structural variations, which makes it critical to understand species evolution through the lens of morphological differentiation. Additionally, the size of the perianth is often closely related to the successful reproduction of plants, and the perianth area is generally considered one of the indicators of perianth size. The Montgomery equation (ME) hypothesizes that the individual leaf area is proportional to the product of leaf length and width, with the proportionality coefficient referred to as the Montgomery parameter (MP). To test the validity of the ME for calculating the tepal area, a total of 541 tepals (including petaloid and sepaloid tepals, which have similar shapes but different colors) from 60 *Liriodendron* × *sinoamericanum* P.C. Yieh ex C.B. Shang & Z.R. Wang flowers were used to fit the relationship between the tepal area (*A*) and the product of the tepal length (*L*) and width (*W*). Furthermore, this study compared whether there were significant differences in MPs between the two types of tepals, as well as differences in the fitting performance of the ME for each type. The root-mean-square error (RMSE) and mean absolute percentage error (MAPE) were used to assess the goodness of fit. The results revealed that the ME had low RMSE values (<0.05) and MAPE values (<5%), along with a high correlation coefficient (>0.95), when fitting the relationship between *A* and *LW* for either of the two different types of tepals. These findings indicate that the ME is effective in predicting the tepal area. Furthermore, there was a difference between the MPs of the two types of tepals. However, since the ME fitting of the data for each tepal type individually, as well as the combined data, all yielded a good fitting performance, the difference between the two types of tepals can be considered negligible in terms of its impact on the fitting results. Therefore, based on the combined morphology and ME fitting results of the two types of tepals, the tepals in *L*. × *sinoamericanum* do not show obvious differentiation. This study provides new insights into the understanding of the differentiation of similar organs during the evolution of angiosperms.

## 1. Introduction

Flowers represent the distinctive sexual reproductive structures of angiosperms, incorporating anatomical features hypothesized to be evolutionarily derived from modified leaves, a perspective extensively substantiated by genetic and evolutionary developmental biology research [1]. Among them, the perianth typically consists of the two outermost whorls of non-reproductive organs in a complete flower. Sepals, leaf-like and usually green, form the first whorl and serve as the external protective layer for the developing bud, whereas petals constitute the second whorl, which are often larger and more colorful [2,3,4,5]. Although these structures are not reproductive, they play critical roles in attracting pollinators and facilitating successful reproduction by serving as visual attractants/guides, nectar producers, protective coverings, and landing platforms for pollinators [6,7]. Plants signal the receptivity of their reproductive organs by flowering and attract pollinators by synthesizing and releasing odors through their petals [8,9]. Consequently, these structures exhibit great diversity in size, shape, and color; thus, they serve as useful models for the study of plant organ development and evolution [10,11].

Most extant flowers (72%) have differentiated perianth arranged within two whorls: the calyx (overall structure of all sepals) and the corolla (collective petal structure), which generally enable optimal organization and structural efficacy [12]. However, in certain plant groups, the sepals and petals are not morphologically distinct and instead appear similar in form and color. In such cases, these undifferentiated, petaloid components are collectively referred to as tepals, as seen in families such as Magnoliaceae, Liliaceae, and Amaryllidaceae [13].

Nevertheless, the outer tepals of some plants are distinctly different in color from those of the inner whorl, which leads to the common misconception that green outer whorl tepals are sepals, (e.g., *Liriodendron* × *sinoamericanum* P.C. Yieh ex C.B. Shang & Z.R. Wang). To our knowledge, no studies have conclusively demonstrated whether the tepals of *L.* × *sinoamericanum* have differentiated into petals and sepals. Therefore, clarifying this issue is crucial for the phylogenetic study of angiosperms and contributes to breeding efforts aimed at improving ornamental traits in *L*. × *sinoamericanum*. Additionally, the size of the perianth is often closely related to the successful reproduction of plants, and perianth area is generally considered one of the indicators of perianth size [14,15]. Thus, it is essential to quantify the size of the perianth (e.g., its area).

The Montgomery equation (ME) estimates the individual leaf area by assuming that it is proportional to the product of leaf length and width [16]. Currently, the ME has been shown to be effective for estimating the areas of leaves, tepals, petals, and stomata, and it is an extensively employed mathematical model in botanical research [17,18,19,20]. Therefore, this study also used ME to fit the tepal area of *L*. × *sinoamericanum*, aiming to evaluate the applicability of this model in estimating the tepal area of this species. Furthermore, Wang et al. [21] found that the ME-fitted parameters (denoted as the Montgomery parameter, MP) for tepals and leaves were very similar in *Magnolia* × *soulangeana*. This provided metrological evidence of floral and foliar homology in plants. Meanwhile, it also suggested that for different plant organs, the relatives were more similar to each other in their MP values. The application of mathematical modeling to test perianth differentiation into sepals and petals represents a novel approach. We selected the ME for this purpose because it has been extensively validated for predicting the area of leaves and perianth organs (including petals and tepals) [17,18,19,20,21]. Compared with other shape-describing models, such as Fourier analysis or the Gielis equation [17,22], the ME is more straightforward, and its effectiveness in botanical studies has been better validated. Thus, from a morphological perspective, the use of ME to examine species in cases where it is unclear whether the petals and sepals of plants are differentiated may provide valuable insights.

**Figure 1:**
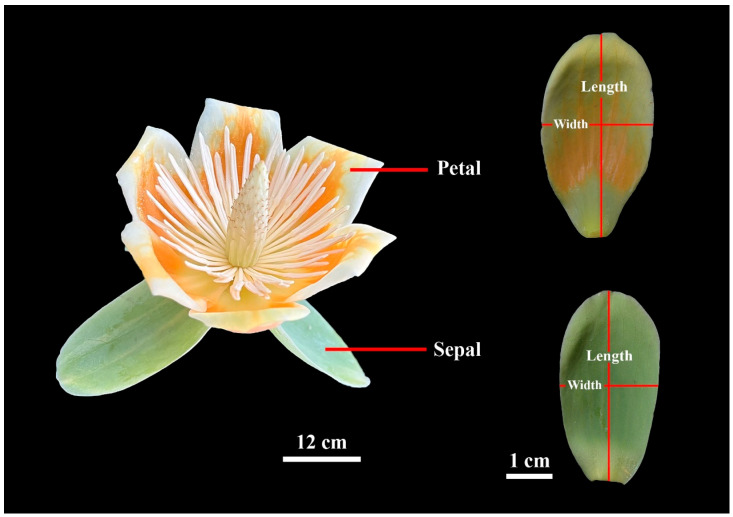
Example of *Liriodendron × sinoamericanum* flower (**left**), and definitions of petal and sepal lengths and widths (**right**).

To investigate these questions, we selected *L.* × *sinoamericanum* for our experiment, which is a hybrid of *Liriodendron chinense* (Hemsl.) Sarg. and *Liriodendron tulipifera* L. This species has large flowers, with three green sepaloid tepals in the first whorl (for convenience, they will hereafter be referred to as sepals), while the six tepals in the second and third whorls (for convenience, they will hereafter be referred to as petals; Figure 1) are orange and have high ornamental value. We sampled 60 flowers in April 2024 when most flowers were fully blooming. And we measured petal area, length, and width of each flower. The objectives of this study were to (i) evaluate the validity of the ME for estimating the individual tepal area, and (ii) to explore tepal differentiation in this species from both a morphological and a mathematical modeling perspective.

## 2. Materials and Methods

### 2.1. Flower Collection Information

A total of 60 pristine blooming flowers of *L.* × *sinoamericanum* were collected on 30 April 2024. The *L.* × *sinoamericanum* trees from which the flowers were sampled were growing naturally at Nanjing Forestry University Xinzhuang Campus in Nanjing, China (32°4′43″ N, 118°48′44″ E). To ensure that the sampled flowers were representative and random, we collected fully bloomed flowers from trees of different sizes with the diameter at breast height (DBH) ranging between 25.7 cm and 45.6 cm. To minimize water loss during transport, the flowers were carefully placed in a foam box (54 × 39.5 × 30 cm^3^) with ice bags and transferred to the laboratory within 1 h.

### 2.2. Data Acquisition

A total of 180 sepals and 361 petals were obtained from 60 randomly selected flowers (one of the flowers had 10 tepals in total, consisting of 7 petals and 3 sepals). Each tepal was scanned at 600 dpi and saved as a bitmap image using a photo scanner (V550, Epson Indonesia, Batam, Indonesia). These images were then converted into black–white .bmp format using Adobe Photoshop 2021 (version 22.0; Adobe, San Jose, CA, USA). During the process, we first used the Object Selection Tool to automatically identify the outlines of the tepals, followed by manual correction of inaccurately identified areas using the Lasso Tool. The MATLAB (version  ≥  2009a; MathWorks, Natick, MA, USA) procedure developed by Shi et al. [22] and Su et al. [23] was utilized to obtain the planar coordinates of the tepal boundary points. The tepal area (*A*), tepal length (*L*), and tepal width (*W*) were calculated using the “bilat” function of the “biogeom” package (version 1.3.5) [24] based on the statistical software R (version 4.3.1; R Core Team, 2023). In this study, *L* was defined as the distance from its apex to base, while *W* was defined as the maximum distance between any two points on the tepal edge perpendicular to the tepal length axis (Figure 1). Further, the centroid ratio was defined as the distance from the tepal base to the point on the tepal length axis associated with the maximum tepal width, divided by the total length [21].

### 2.3. Statistical Analyses

The coefficient of variation (CV) was employed to describe the relative dispersion of the tepal size and shape:

(1) $$\mathrm{CV}=\frac{\sigma }{\mu }\times 100\%,$$

where *σ* is the standard deviation of the tepal data and *μ* is the mean of the tepal data. The smaller the CV value, the smaller the variation.

Student’s *t*-test [25] was used to determine whether there were significant differences in sizes (i.e., tepal area, tepal length) and shapes (i.e., ratio of tepal width to length, and tepal centroid ratio) between the sepals and petals.

The ME, first proposed by Montgomery [16] for corn leaves, postulates that leaf area is proportional to the product of leaf length and width. In the present study, the ME was used to test whether the tepal area (*A*) had a proportional relationship with the product of the tepal length (*L*) and width (*W*), i.e.,

(2) A=kLW,

where *k* is the proportionality coefficient, which is referred to as the Montgomery parameter (MP) [16,17,18,19].

To stabilize variance, the two sides of Equation (2) were logarithmically transformed, i.e.,

(3) ln(A)=ln(k)+ln(LW),

Least-squares regression protocols were used to minimize the residual sum of squares to estimate the model parameters. The 95% confidence intervals (CIs) for the MPs were obtained using the bootstrap percentile method [26,27] with 3000 bootstrap replicates. We tested whether the 95% confidence interval (CI) of the differences between the MP replicates for sepals and petals included 0. If so, this indicated no significant difference; otherwise, it did exist.

The root-mean-square error (RMSE) was calculated to assess the goodness of fit of the linear regression:

(4) $$\mathrm{RMSE}=\sqrt{\frac{\sum_{i=1}^{n}{\left\{\log\left(A_{i}\right)-\log\left({\hat{A}}_{i}\right)\right\}}^{2}}{n}},$$

where log(*A_i_*) is the natural logarithm of the observed (scanned) area of the *i*th tepal, log(*Â_i_*) is the natural logarithm of the predicted area of the *i*th tepal using the ME, and *n* represents the number of tepals. If the RMSE value was smaller, the goodness of fit was better. Furthermore, the correlation coefficient (*r*) was used to measure the strength of the linear correlation between log(*A*) and log(*LW*).

The mean absolute percentage error (MAPE) was used to evaluate the effectiveness of the model in predicting the tepal accuracy, i.e.,

(5) $$\mathrm{MAPE}=\frac{1}{n}\sum_{i=1}^{n}\left|\frac{A_{i}-{\hat{A}}_{i}}{A_{i}}\right|\times 100\%,$$

where *A_i_* is the observed (scanned) area of the *i*th tepal, *Â_i_* is the predicted area of the *i*th tepal using the ME, and *n* represents the number of tepals. A smaller MAPE value indicated that the prediction error of ME was smaller and closer to its true value.

## 3. Results

**Figure 2:**
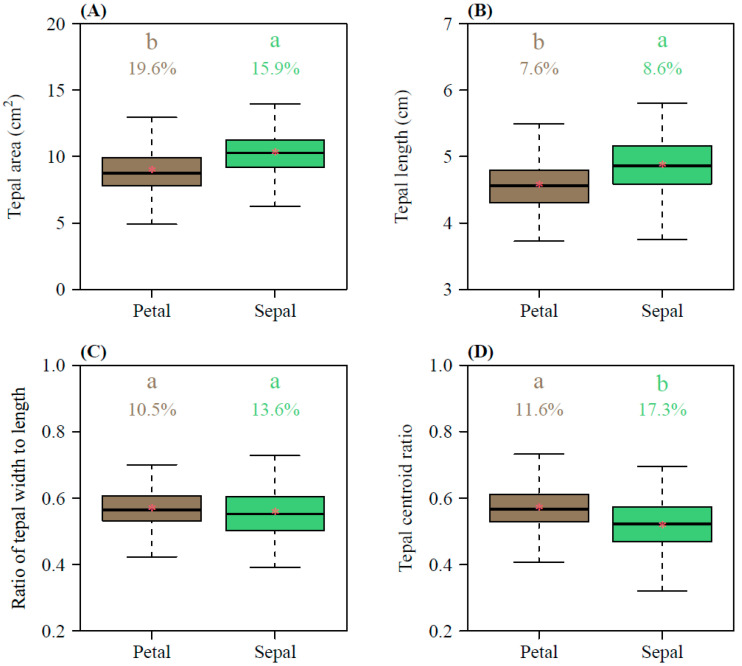
Comparisons of the tepal area (**A**), tepal length (**B**), tepal width/length ratio (**C**), and tepal centroid ratio (**D**) between petals and sepals. The lowercase a and b at the top of each box denote the significance of the difference in the means between petals and sepals based on Student’s *t*-test at a 0.05 significance level. Numbers above the whiskers represent the coefficients of variation (%). Horizontal bold lines in the boxes represent medians, and asterisks within the boxes represent means.

The petal areas ranged from 4.91 to 14.09 cm^2^, with a mean of 8.93 ± 1.67 (Mean ± SD) cm^2^, whereas the sepal areas ranged from 6.26 to 15.35 cm^2^, with a mean of 10.30 ± 1.63 cm^2^ (Figure 2A); the petal lengths ranged from 3.73 to 5.63 cm, with a mean of 4.58 ± 0.35 cm, whereas the sepal lengths ranged from 3.62 to 6.21 cm, with a mean of 4.87 ± 0.42 cm (Figure 2B). Overall, the petals were smaller in both area and length than the sepals, and this difference was statistically significant (*p* < 0.01; Figure 2A,B). In terms of the tepal shape, the width/length ratios of the petals ranged from 0.419 to 0.700, with a mean of 0.568 ± 0.056 cm, and the width/length ratios of the sepals ranged from 0.391 to 0.775, with a mean of 0.558 ± 0.076 (Figure 2C). There was no significant difference between the two types of tepals (*p* = 0.1061; Figure 2C). The petals had a slightly larger centroid ratio (0.57 ± 0.07) than the sepals (0.52 ± 0.09), with a statistically significant difference between the two (*p* < 0.01; Figure 2D). Except for the tepal area, the coefficients of variation in the petal length, petal width/length ratio, and petal centroid ratio were significantly lower than those of the sepals (Figure 2).

**Figure 3:**
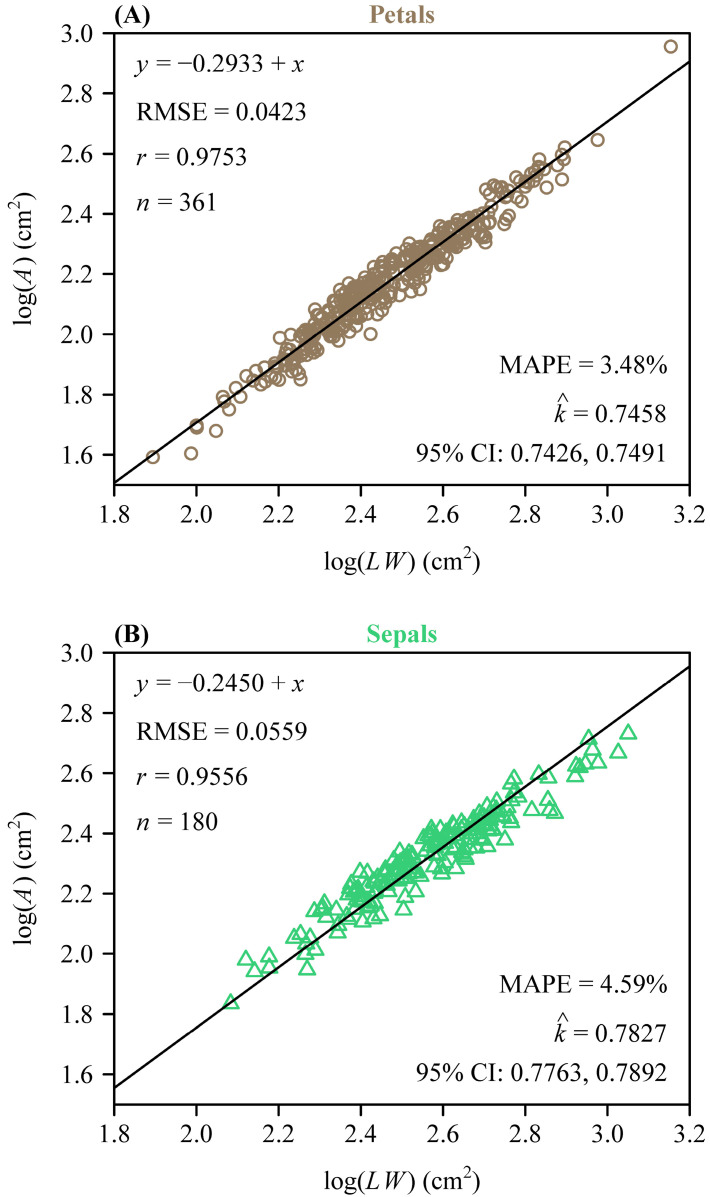
Results of fitting the ME on a log–log scale for petals (**A**), and sepals (**B**). In each panel, *A*, *L*, and *W* represent the tepal area, length, and width, respectively; RMSE represents the root-mean-square error for the linear fitting; *r* is the correlation coefficient; *n* is the number of tepals; $\hat{k}\;$denotes the estimated Montgomery parameter; MAPE is the mean absolute percentage error between the observed and predicted areas; CI represents the 95% confidence interval of the MP based on 3000 bootstrap replicates. The symbols are observations (open circles in panel (**A**): petals; open triangles in panel (**B**): sepals), and the straight line is the regression line predicted by the Montgomery equation on a log-log scale.

**Figure 4:**
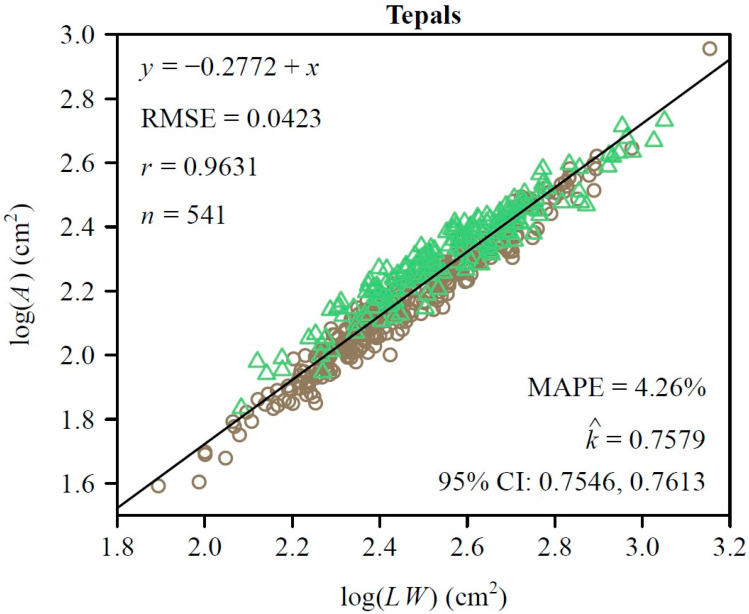
Results of fitting the ME on a log–log scale for the pooled data of the two types of tepals. In the panel, *A*, *L*, and *W* represent the tepal area, length, and width, respectively; RMSE represents the root-mean-square error for the linear fitting; *r* is the correlation coefficient; *n* is the number of tepals; $\hat{k}\;$ denotes the estimated MP; MAPE is the mean absolute percentage error between the observed and predicted areas; CI represent the 95% confidence interval of the MP based on 3000 bootstrap replicates. The symbols are observations (open circles: petals; open triangles: sepals), and the straight line is the regression line predicted by the Montgomery equation on a log-log scale.

To further quantify morphological similarity, we assessed the applicability of the Montgomery equation (ME) to the two types of tepals. For both sepals and petals, there was a significant proportional relationship between their areas and the products of their lengths and widths. The estimated MP value for the petals was 0.7458 (Figure 3A) and for the sepals was 0.7827 (Figure 3B). The RMSE values of the linear regression were 0.0423 for the petals and 0.0559 for the sepals, while the correlation coefficients were 0.9753 and 0.9556, respectively (Figure 3A,B). This validated the ME in describing the *A* and *LW* relationship of *L.* × *sinoamericanum*. Furthermore, the 95% CIs (i.e., −0.058, −0.039) of the difference between the two groups of MP replicates did not include 0, which indicated that there was a significant difference between the two MPs. However, at the combination data level, the RMSE of the log of observed area versus the log of predicted area for the tepals was <0.05, and the MAPE values were also <5% (Figure 4). This indicated that the model achieved accurate predictions with minimal errors, which suggested that the effect of this discrepancy on the study results was negligible and could be ignored. Overall, these results suggest that petals and sepals in *L*. × *sinoamericanum* differed significantly in several morphological traits, whereas they both validated the ME in calculating tepal area.

## 4. Discussion

### 4.1. Effectiveness of the Montgomery Equation in Calculating the Tepal Area

Many studies have demonstrated that the ME is a simple yet effective tool for estimating leaf areas, applicable across different shapes and sizes [17,18,19,28,29]. Subsequently, Zhang et al. [30] evaluated the effectiveness of ME in calculating leaf stomatal areas, and Wang et al. [21] demonstrated its effectiveness in predicting the tepal areas of *M.* × *soulangeana*. In the present study, we applied the ME to fit the tepal area of *L.* × *sinoamericanum*. The fitted results (RMSE < 0.05, MAPE < 5%) also indicated that the ME was reliable for predicting the areas of foliar homologue (i.e., tepals) (Figure 4). It is worth noting that whether fitting the areas of petals and sepals separately or combining them into a single dataset, the results were highly accurate in all cases, which further supported the above conclusion.

However, due to the significant morphological diversity of tepals, petals, and leaves spanning different plant species, including variations in their sizes, shapes, and complex surface structures, the effectiveness of the ME in calculating plant organ areas warrants further investigation [31,32,33,34,35,36].

### 4.2. Exploration of the Montgomery Parameter in Quantifying Tepal Shapes

Shi et al. [37] sampled the leaves of two *Liriodendron* species (i.e., *L. chinense* and *L. tulipifera*) and their hybrid (*L.* × *sinoamericanum*), and obtained an MP estimate of 0.7198 for the three datasets. In this study, the MP estimate for the tepals of *L.* × *sinoamericanum* was 0.7579. Although the MP of tepals was slightly greater than that of leaves, the two values were relatively approximate. This difference may have been due to the variations in shape between the leaves and tepals of this species, as well as the fact that the tepals were not as flat as the leaves. Leaves are typically flatter, which enhances the acquisition of light, whereas tepals generally exhibit some degree of curvature, and it is intimately associated with their protective function for the fragile internal stamen and pistil. Consequently, we posited that this difference was the result of plant adaptations and evolution, while their similarities supported the concept of floral and foliar homology [38,39].

Despite the statistically significant differences in the MP values between the two types of tepals (petals and sepals), these discrepancies had negligible practical impacts, as evidenced by the low RMSE and MAPE metrics (RMSE < 0.05, MAPE < 5%) for the pooled data. Therefore, when comparing the MP values of the two, the data indicate that the two types of tepals did not exhibit a clear differentiation. Although the sepals were larger in size (in terms of area and length) than the petals (Figure 2A,B), there was no significant difference in their tepal width/length ratios (Figure 2C). Instead, the large size of the sepals may have been due to their role of wrapping around the entire flower to protect the internal structure prior to flowering [40].

Nevertheless, He et al. [41] found that between different plant families, completely distinct leaf shapes can result in similar MP values. This may be because the ME assumes a specific proportionality between the leaf area and the product of leaf length and width. When the planar projections of organic structures have closely similar shapes, this proportional relationship may not fully capture the subtle differences in size or structure. That is to say, in spite of the fact that MP offers a useful comparative metric, its reliability is likely to diminish in structures with complex or non-elliptical geometry. Therefore, models that possess more complex structures might be used in the future to capture the features of similarly shaped tepals to facilitate more refined comparisons.

### 4.3. Evolution of Perianth Differentiation in Magnoliaceae: Insights from Morphometrics and Gene Expression

Magnoliaceae represents one of the basal lineages of angiosperms, which is often characterized by undifferentiated tepals, where the petals and sepals are morphologically similar [42,43,44,45]. According to comparative morphology, most researchers agree that sepals evolved from bracts or leaves, while petals may have evolved from either bracts or stamens. However, in some groups, sepals and petals are considered to be homologous structures as both originate from bracts or leaves [46,47,48,49].

In this study, *L.* × *sinoamericanum* exemplified this undifferentiated perianth structure, despite color differences between the sepal (green) and petal (orange), their morphological parameters (e.g., tepal width/length ratio) (Figure 2C), and the ME-based fitted results (Figure 3A,B), which showed minimal divergence. This supported the lack of clear differentiation between the sepals and petals. These findings aligned with the ancestral state of Magnoliaceae, where tepals retain a transitional morphology between protective bracts and specialized petals [50]. These findings can be interpreted through several developmental models of floral organ identity. This pattern may be closely related to the mechanisms proposed by the Sliding Boundary and Fading Boundary models [51,52]. According to the Sliding Boundary model, the expansion of B-class gene expression (e.g., *AP3* and *PI*) could lead to the morphological convergence of sepals and petals. Such gene expression patterns may reduce the extent of morphological divergence between the sepals and petals, which allow the tepals to retain the transitional characteristics of ancestral floral organs [53,54]. Conversely, the Fading Boundary model suggests that the gradient patterns of gene expression might translate to the partial differentiation of traits such as color, rather than the formation of entirely distinct organs [55]. Such gradient patterns allow tepals to achieve partial functional specialization without complete differentiation into sepals or petals, thereby maintaining morphological homogeneity.

Furthermore, the Mosaic theory suggests that the distinction between sepalness and petalness evolved early in angiosperm history. However, these features were not fixed to specific organs and primarily environmentally controlled [48]. In *L.* × *sinoamericanum*, green sepals may be associated with protective functions, while orange petals may serve to attract pollinators [56]. This color differentiation may be induced by the gradient expression of pigment synthesis genes (e.g., those involved in flavonoid or carotenoid pathways) across different whorls of tepals [57,58]. This aligned with the genetic evidence observed in Magnoliaceae, where the perianth elements exhibited flexibility in their morphological characteristics based on gene expression patterns and environmental influences.

In addition, our findings support the hypothesis that tepals in basal angiosperms such as Magnoliaceae retain ancestral features shaped by gradients in gene expression rather than strict organ identity. These findings underscore the importance of basal lineages in the study of floral evolution and offer new perspectives on the origin and diversification of floral diversity in angiosperms.

## 5. Conclusions

The data show that the Montgomery equation is valid for calculating the tepal area of *L.* × *sinoamericanum* by assuming that the organ area is proportional to the product of the tepal length and width. Furthermore, by comprehensively comparing the Montgomery parameter values that were used to detect the probable morphological variations between petals and sepals, we conclude that the two types of tepals are not clearly differentiated. This study provides new insights into the understanding of the differentiation of similar organs during the evolution of angiosperms. Additionally, we suggest the use of more structurally complex models in the future (e.g., *L.* × *sinoamericanum*) to study species with similar tepal traits, aimed at deriving more refined results that can contribute to a better understanding of their evolution.

## Data Availability

The data can be found in the online Supplementary Table S1.

## Supplementary Materials

The following supporting information can be downloaded at https://www.mdpi.com/article/10.3390/plants14121861/s1, Table S1: Raw data of tepal area, length, and width of *Liriodendron* × *sinoamericanum*.
